# Designer exosomes for targeted and efficient ferroptosis induction in cancer via chemo-photodynamic therapy

**DOI:** 10.7150/thno.59121

**Published:** 2021-07-13

**Authors:** Jianbing Du, Zhuo Wan, Cong Wang, Fan Lu, Mengying Wei, Desheng Wang, Qiang Hao

**Affiliations:** 1 Department of Hepatobiliary Surgery, Xijing Hospital, Fourth Military Medical University, Xi'an, 710032, China.; 2 State Key Laboratory of Cancer Biology, Biotechnology Center, School of Pharmacy, Fourth Military Medical University, Xi'an, 710032, China.; 3 Department of Hematology, Tangdu Hospital, Fourth Military Medical University, Xi'an, 710038, People's Republic of China.; 4 Department of Clinical Laboratory, The Second People's Hospital of Hefei, Hefei, China.; 5 State Key Laboratory of Cancer Biology, Department of Biochemistry and Molecular Biology, Fourth Military Medical University, Xi'an, 710032, People's Republic of China.

**Keywords:** Hepatocellular carcinoma, exosomes, ferroptosis, photodynamic therapy, synergistic effects

## Abstract

**Background:** Efficient and specific induction of cell death in liver cancer is urgently needed. In this study, we aimed to design an exosome-based platform to deliver ferroptosis inducer (Erastin, Er) and photosensitizer (Rose Bengal, RB) into tumor tissues with high specificity.

**Methods:** Exosome donor cells (HEK293T) were transfected with control or CD47-overexpressing plasmid. Exosomes were isolated and loaded with Er and RB via sonication method. Hepa1-6 cell xenograft C57BL/6 model was injected with control and engineered exosomes via tail vein. *In vivo* distribution of the injected exosomes was analyzed via tracking the fluorescence labeled exosomes. Photodynamic therapy was conducted by 532 nm laser irradiation. The therapeutic effects on hepatocellular carcinoma and toxic side-effects were systemically analyzed.

**Results:** CD47 was efficiently loaded on the exosomes from the donor cells when CD47 was forced expressed by transfection. CD47 surface functionalization (Exos^CD47^) made the exosomes effectively escape the phagocytosis of mononuclear phagocyte system (MPS), and thus increased the distribution in tumor tissues. Erastin and RB could be effectively encapsulated into exosomes after sonication, and the drug-loaded exosomes (Er/RB@Exos^CD47^) strongly induced ferroptosis both *in vitro* and *in vivo* in tumor cells after irradiation of 532 nm laser. Moreover, compared with the control exosomes (Er/RB@Exos^Ctrl^), Er/RB@Exos^CD47^ displayed much lower toxicity in liver.

**Conclusion**: The engineered exosomes composed of CD47, Erastin, and Rose Bengal, induce obvious ferroptosis in hepatocellular carcinoma (HCC) with minimized toxicity in liver and kidney. The proposed exosomes would provide a promising strategy to treat types of malignant tumors.

## Introduction

Despite considerable progress in diagnosis and therapy, primary liver cancer ranks the fifth leading cause of cancer death 1. Currently, beyond surgical resection, the treatments are mainly chemotherapy, targeted drug therapy and immunotherapy 2-4. However, the effectiveness of these therapies in cancer is limited 5, 6. Therefore, it is particularly urgent to explore other strategies to induce cell death specific in cancer.

Recent years, ferroptosis has attracted the attention of many researchers in the field of tumor research 7. Ferroptosis is a new form of cell death first proposed in 2012, which has been accepted as a novel type of feature to eliminate tumor cells 8, 9. Ferroptosis depends on iron-dependent Fenton reaction and the accumulation of lipid peroxides, is totally different from necrosis and apoptosis 10. The characteristic of ferroptosis is the exhaustion of the intracellular pool of glutathione (reduced) (GSH), passivation of the glutathione peroxidase 4 (GPX4), eventually accumulation of lipid reactive oxygen species (ROS) 11. The cystine-glutamate antiporter (system x_c_^-^), a critical transporter regulates glutamate homeostasis, plays a great role in ferroptosis 10, 12. Existing evidence have showed ferroptosis played a therapeutic role in many apoptosis-resistant tumors, such as gastric cancer, pancreatic cancer, colorectal cancer and HCC 13-16. Therefore, inducing tumor cell ferroptosis will open a new therapeutic avenue for hepatocellular carcinoma (HCC).

In 2012, researchers defined the form of cell death caused by Erastin as ferroptosis 10. Erastin, as a classic ferroptosis inducer, has been explored in the treatment of tumors which can directly inhibit the activity of the system x_c_^-^ 10, 17. However, free Erastin has low water solubility and high nephrotoxicity, limiting its application 18. In addition, locally induced ROS leads to the accumulation of lipid ROS in cells, which will promote ferroptosis and thus reduced the need of Erastin. Photodynamic therapy (PDT) is a technology that irradiating a specific wavelength light to excite the photosensitizer (PS) in the target site to result in a significant accumulation of ROS 19. Rose Bengal (RB) is a famous photosensitizer which has a high ROS yield in PDT 20. Therefore, efficiently delivering Erastin and RB to tumor tissues might synergistically induce efficient ferroptosis with low side effects is a promising method to treat cancer.

In the past decade, the research of drug delivery nanocarriers have been rapidly developed 21. However, their application has been limited due to several reasons, such as, low load capacity, biodistribution problem and cationic ligand toxicity 22, 23. Exosome, a small nanovesicle, is a new type of communication and drug delivery medium that can transport biologically active molecules between cells through a variety of biological molecules (such as proteins and nucleic acid) and regulates cells microenvironment and immune system 24-27. Unlike other nanoparticle carriers, exosomes contain transmembrane proteins that may enhance endocytosis, thus promoting the delivery of their internal content 28. As a delivery carrier, exosomes have the advantages of low immunogenicity, high biocompatibility and enhanced permeability and retention effect in tumor issues 29. However, most injected nanomaterials are phagocytized by the mononuclear phagocyte system (MPS, e.g., liver and spleen), preventing their delivery to the desired area 30-33. CD47, which is a widely expressed integrin-related transmembrane protein, is the ligand for signal regulatory protein alpha (SIRPα), and CD47-SIRPα binding initiates the “don't eat me” signal that inhibits phagocytosis 28, 34-36. Engineering of CD47 on the exosomes could protect the exosomes from phagocytosis by macrophages.

In this study, we have engineered an exosome armed with three moieties, surface functionalization with CD47, membrane loading with ferroptosis inducer Erastin, and core with photosensitizer RB. The exosomes displayed high delivery efficiency to tumors. Upon irradiation of 532 nm laser in the tumor region, Er and RB synergistically induced cell death. By using the HCC xenograft model, we here showed that the designed exosomes display potent anti-tumor therapeutic effects, and amazing low toxicity, which would provide a promising strategy to treat malignant tumors.

## Materials and methods

### Cell culture

HEK293T, RAW264.7 cells (obtained from ATCC) and Hepa1-6 cells (carried luciferase reporter gene) (donated by Pro. Dou from the Fourth Military Medical University) were maintained in Dulbeccoo's modified Eagle's medium (DMEM) (Hyclone, USA) supplemented with 10% fetal bovine serum (HyClone, USA), 1% penicillin/streptomycin (HyClone, USA). All cells were cultured at 37 °C in 5% CO_2_.

### Tumor-bearing models

Male C57BL/6 mice (6-8 weeks) were purchased from the animal laboratory center of the Fourth Military Medical University. All animal experiments were approved by the Animal Experiment Administration Committee of the Fourth Military Medical University. Hepa1-6 cells (3×10^6^ cells in 100 μL phosphate buffered saline, PBS) were subcutaneously injected on the left back of mice for subcutaneous HCC model. The long diameter and short diameter of tumor were measured by vernier caliper. Tumor volume was estimated as (L × S^2^) × 0.5 (L, long diameter; S, short diameter).

### Plasmid construction

CD47 was amplified with cDNA in Hepa1-6 cells by PCR using primers flanking with corresponding enzyme sites. Then the amplicons were inserted between BamH I and Hind III into a pcDNA3.1(-) vector, with the resultant correct plasmids designated as pcDNA 3.1(-)-CD47. All the clones were confirmed by sequencing and the right clones were stored at -80 °C for following application.

### Transfection

To produce exosomes engineered with CD47, HEK293T cells were transfected with the corresponding plasmid with Lipofectamine 2000 (Invitrogen) as instructed. Briefly, when the HEK293T cells density grew to 60%-70% in 100-mm culture dish, culture medium was replaced with antibiotic-free medium. Then, 5 μg plasmid and 10 μL Lipofectamine 2000 were added to two centrifuge tubes containing 1 mL of DMEM medium respectively and incubated at room temperature for 5 min. Then, the contents of the two centrifuge tubes were mixed together and incubated at room temperature for 5 min. The above mixed culture medium was added to the 293T cell culture dish.

### Exosome preparation

Exosomes were extracted from the culture supernatant of HEK293T. HEK293T cells were washed twice with PBS and replaced with serum-free medium. After the HEK293T cells were cultured in a serum-free medium at 37 °C in 5% CO_2_ for 48 h, the supernatant was collected. Exosomes were isolated by ultracentrifugation. Briefly, the culture medium was centrifuged at 3000 g for 15 min to remove cell debris, and the resulting supernatant was filtered using a 0.22 μm filter. Then the supernatant was ultracentrifuged at 100,000 g for 60 min and then the pellet was resuspended and repeated again. The resulting precipitate, namely exosomes, was diluted in PBS and stored at -80 ℃ until use.

### Exosome characterization

The concentration of exosomes was measured by total proteins using the Pierce BCA Protein Assay Kit (Thermo Scientific, USA). The exosomes were diluted to 1 mg/mL, and the size distribution was analyzed by NanoPlus (Otsuka Electronics, Japan). The morphology of exosomes was observed by transmission electron microscopy (JEM-2000EX TEM, Japan). The expression of exosome proteins of TSG101, CD9 and CD47 were determined by western blot.

### Exosome pulldown assay

For analysis of the expression of CD47 on exosomes, we performed exosome pull down assay using anti-CD47 (Abcam, ab175388) antibodies as described before 37. Briefly, about 50 μL of Protein-A Sepharose beads in 0.2% BSA in 1 mL of PBS was first incubated with 5 μg of anti-CD47 or control IgG at 4 °C overnight. The unbound anti-CD47 was removed by centrifugation. Then, the antibody bound Protein-A Sepharose beads were incubated with the exosomes, followed by precipitation. The immunoprecipitated exosomes were then subjected to lysis buffer, followed by Western blotting assay.

### Western blotting

Protein was extracted from exosomes, HEK293T cells, Hepa1-6 cells or tumor tissues, lysed with RIPA lysis buffer at 4 °C for 30 min, and then the protein concentration was measured using Pierce BCA protein kit (Thermo, USA). Protein samples were concentrated on 5% SDS-PAGE gel and separated by 12% SDS-PAGE gel. After that, the gel was transferred to a nitrocellulose filter membrane. After blocking with 5% BSA, NC membrane was incubated with primary antibody overnight at 4 ℃. The corresponding HRP-conjugated secondary antibodies were incubated for 1 h at room temperature and then the band was visualized by ECL tool (GE Healthcare, UK). Antibodies used were mouse anti-TSG101 (1:200, Santa, sc-7964), rabbit anti-CD9 (1:2,000, Abcam, ab92726), rabbit anti-GM130 (1:1,000, Abcam, ab30637), rabbit anti-CD47 (1:1,000, Abcam, ab175388), mouse anti-GPX4 (1:200, Santa, sc-166570), mouse anti-GAPDH (1:20,000, Proteintech, 60004-1-1g). The secondary antibodies used were horse anti-mouse (1:2,000, Cell Signaling Technology, 7076P2), goat anti-rabbit (1:2,000, Cell Signaling Technology, 7074P2).

#### Cel-miR-54 mimics loading into exosomes

Exosomes at a protein concentration of 0.5 mg/mL (0.8 mL) (BCA Protein Assay Kit, Thermo Scientific) were electroporated with 0.5 OD cel-miR-54 mimics (GenePharma, Shanghai, China) in 4 mm cuvette at 700 V and 50 μF for 3 pulses. Following electroporation, exosomes were treated with RNase to remove miRNAs that had not been loaded into the exosomes. Then, exosomes were washed with PBS and ultracentrifuged at 100,000 g for 60 min.

### Quantitative real-time PCR for exosome phagocytosis *in vitro*

The exosomes (100 μg) loaded with cel-miR-54 mimics were added to Hepa1-6 cells or RAW264.7 cells cultured in 100-mm culture dish respectively. Then, the cells and exosomes were incubated for 6 h. Total RNA from exosomes or cells was extracted by Trizol (Invitrogen) following the manufacturer's instructions. The concentration of RNA was measured with a Nanodrop-2000 spectrophotometer (Thermo Scientific, USA). For analysis of miRNA expression, 2 μg total RNA was reverse transcribed using miRcute Plus miRNA First-Strand cDNA Kit (KR211-02, TIANGEN, China) and qPCR was performed using FastStart Essential DNA Green Master Kit (06924204001, Roche). U6 was used as an internal control. All kits were used following the manufacturers' instructions. The primer sequences are listed in Table S1.

### Quantitative real-time PCR for exosome phagocytosis in the circulation

The exosomes (about 200 μg) loaded with cel-miR-54 mimics were injected into the mice via the tail vein. 2 h and 24 h after injection, blood was collected by removing eyeball and the serum was separated by 3000 g, 5 min centrifuge. Total RNA from the serum was extracted by Trizol. Then the expression of cel-miR-54 was analyzed as previous described.

### *In vitro* and *in vivo* fluorescence tracing of exosomes

For *in vitro* tracing of exosomes in RAW264.7 cells and Hepa1-6 cells, exosomes (1 μg/μL, 300 μL) were labeled with DiI (Invitrogen, USA) by incubating with the dye (1 mM) at the ratio of 400:1 in volume for 20 min, followed by exosomes isolation as described above. RAW264.7 cells or Hepa1-6 cells cultured in glass bottom cell culture dish were incubated with DiI-labeled exosomes (about 50 μg) for 6 h. The cells were washed with PBS three times and fixed with 4% paraformaldehyde for 10 min and washed with PBS twice. The cells nuclei were counter-stained with 1 μg/mL Hoechst 33342 (C1022, Beyotime, China) for 10 min in dark room at 37 °C. In the end, the cells were washed with PBS for 3 times and observed using a Nikon A1 Spectral Confocal Microscope (Nikon, Japan).

For *in vivo* tracing of exosomes, the exosomes (about 200 μg) were labeled with the fluorescent dye DiR (Invitrogen, USA) by incubating with the dye (1 mM) at the ratio of 400:1 in volume and injected into the tumor-bearing mice via the tail vein to analyze the distribution of exosomes. 6 h later, the mice were sacrificed by cervical dislocation, then the organs and tumors were taken out, and the distribution of fluorescently labeled exosomes in various organs was observed using *in vivo* imaging system (PerkinElmer, USA). To further examine the location of exosomes in different organs and tumors. The exosomes were labeled with the fluorescent dye DiI and injected into the tumor-bearing mice via the tail vein. 6 h later, the mice were sacrificed by cervical dislocation, then, the organs and tumors were taken out. The organs and tumors were stored in the optimal cutting temperature (O.C.T) specimen matrix for cryosections at -20 °C by cryostat, fixed with 4% paraformaldehyde at room temperature for 10 min, washed with PBS three times, and then stained with 1 μg/mL Hoechst 33342 for 10 min. Next, the tissue sections were imaged by Nikon A1 Spectral Confocal Microscope.

### Erastin (Er) and Rose Bengal (RB) loading into exosomes

Erastin (MedChemExpress, USA) and Rose Bengal (Sigma-Aldrich, USA) were added to the exosomes (1 mL) and the mixture was sonicated 38 (20% amplitude, 6 cycles, 10 s on/off, 3 min duration, 2 min cooling period between each cycle) by Ultrasonic Homogenizer (Cole-Parmer, USA). The solution was then incubated at 37 °C for 4 h to restore the exosome membrane to form Er/RB@Exos. Er/RB@Exos was obtained by centrifugation and excess Er and RB were eliminated in the supernatant.

### Determination of loading capacity

Er and RB were respectively dissolved in DMSO and PBS at a concentration of 10 mM and stored at -20 ℃. To determine the maximum drug loading dose of RB in exosomes, different dose of RB was added to the exosomes (600 μg/mL, 1 mL, total protein) (the group of exosomes) and the same dose RB was added to PBS (the group of RB). Then, the UV-vis absorption spectrum of RB was collected by the Nanodrop-2000 spectrophotometer (Thermo Scientific, USA) and the absorption intensity at the maximum absorption peak was used to calculate the drug concentration. For the group of exosomes, after sonication, different mixtures were centrifuged at 100,000 g for 60 min and then the absorption intensity (*A_1_*) of the supernatant at the peak was measured. For the group of RB, the absorption intensity (*A_2_*) of the aqueous solution at the peak was also measured. The loading rate of RB in exosomes were calculated by the following equation: loading rate = (*A_2_*-* A_1_*) / *A_2_*× 100%. The same method was applied to measure the loading rate of Er in exosomes. In the meantime, the UV-vis absorption spectrum of exosomes was collected by the Nanodrop-2000 spectrophotometer.

### Cells experiments

Hepa1-6 cells were cultured in 6-well plates, then different groups of exosomes (about 100 μg) were added to per well and incubated for 6 h and cells without any treatment were used as the control groups. 6 h later, the medium was replaced with fresh medium to remove the rest exosomes that had not been phagocytosed by cells. 6 h later, the light groups were given laser light (532 nm, 10 mW/cm^2^, 5 min).

### MTT assay

Hepa1-6 cells were cultured in 96-well plates, then different groups of exosomes (about 10 μg) were added to per well. 24 h after different treatments, the cell viability was detected by MTT assay according to protocol of MTT kit (ST316, Beyotime, China).

### Flow cytometry analysis for lipid ROS

Hepa1-6 cells were cultured in 6-well plates. Cells were treated and then 50 μM C11-BODIPY 581/591 (MX5211-1MG, MKBio, China) was added and incubated for 1 h. Lipid ROS generation was measured by a flow cytometer according to the method of Gao M, et al 39.

### Animal experiments

Mice were injected with control or engineered exosomes through the tail vein for two consecutive weeks and the light groups were given laser light (532 nm, 100 mW/cm^2^, 15 min), twice a day. At the end of the experiment, the mice were sacrificed and the inoculated tumors were removed, and then the tumor volume was measured and histologically analyzed. From the treatment to the end of the experiment, the size of the subcutaneous tumor was measured every two days using a vernier caliper, and the weight change of the mice was recorded.

### Total ROS detection

Hepa1-6 cells with density of 10^4^ cells per mL were seeded on glass bottom cell culture dish. Treated cells were incubated with 1μM dihydroethidium (DHE) (S0063, Beyotime) for 30 min at 37 °C, fixed with 4% paraformaldehyde at room temperature for 10 min, then washed with PBS three times and subsequently followed by nuclei staining with 1 μg/mL Hoechst 33342 for 10 min. Images were then captured by Nikon A1 Spectral Confocal Microscope (Nikon, Japan). In order to evaluate the production of superoxide histologically, the tumors cut from the mice were stored in the optimal cutting temperature (O.C.T) specimen matrix (SAKURA, USA) for cryosections at 20 °C by cryostat (Leica, USA). Frozen tumor tissue sections (about 8 μm thick) were stained with 1μM dihydroethidium (DHE) at 37 °C for 30 min, washed with PBS three times, fixed with 10% paraformaldehyde at room temperature for 10 min, washed with PBS three times. Then tissue sections were stained with 1 μg/mL Hoechst 33342 for 10 min. The images were observed under Nikon A1 Spectral Confocal Microscope (Nikon, Japan).

### Tumor lipid ROS measurement

In order to evaluate the production of lipid ROS histologically, the tumors cut from the mice were stored in the optimal cutting temperature (O.C.T) specimen matrix (SAKURA, USA) for cryosections at -20 °C by cryostat (Leica, USA). Frozen tumor tissue sections (about 8 μm thick) were stained with 2 μM C11 BODIPY 581/591 at 37 °C for 20 min, washed with PBS three times, fixed with 10% paraformaldehyde at room temperature for 10 min, washed with PBS three times. Then tissue sections were stained with 1 μg/mL Hoechst 33342 for 10 min. The oxidized C11 BODIPY 581/591 were observed under Nikon A1 Spectral Confocal Microscope (Nikon, Japan).

### Cytotoxicity evaluation

Mice were injected with PBS (as control), Er/RB@Exos^Ctrl^ or Er/RB@Exos^CD47^ through the tail vein for two consecutive weeks. Blood was collected by removing eyeball, and then the serum was separated by 3000 g, 5 min centrifuge, which was used to detect alanine transaminase (ALT), aspartate transaminase (AST), blood urea nitrogen (BUN), creatinine (Cr), creatine kinase (CK) and creatine kinase-MB (CK-MB). After the mice were sacrificed by cervical dislocation, the organs of livers and kidneys were taken out for H&E staining.

### Statistical analysis

The data were expressed as the mean ± SEM. Student t-test was used for two group comparison and one-way ANOVA was used for comparison among more than two groups. The differences were considered statistically significant when the *P* value was < 0.05.

## Results

### Engineering and characterization of engineered exosomes

**Figure 1:**
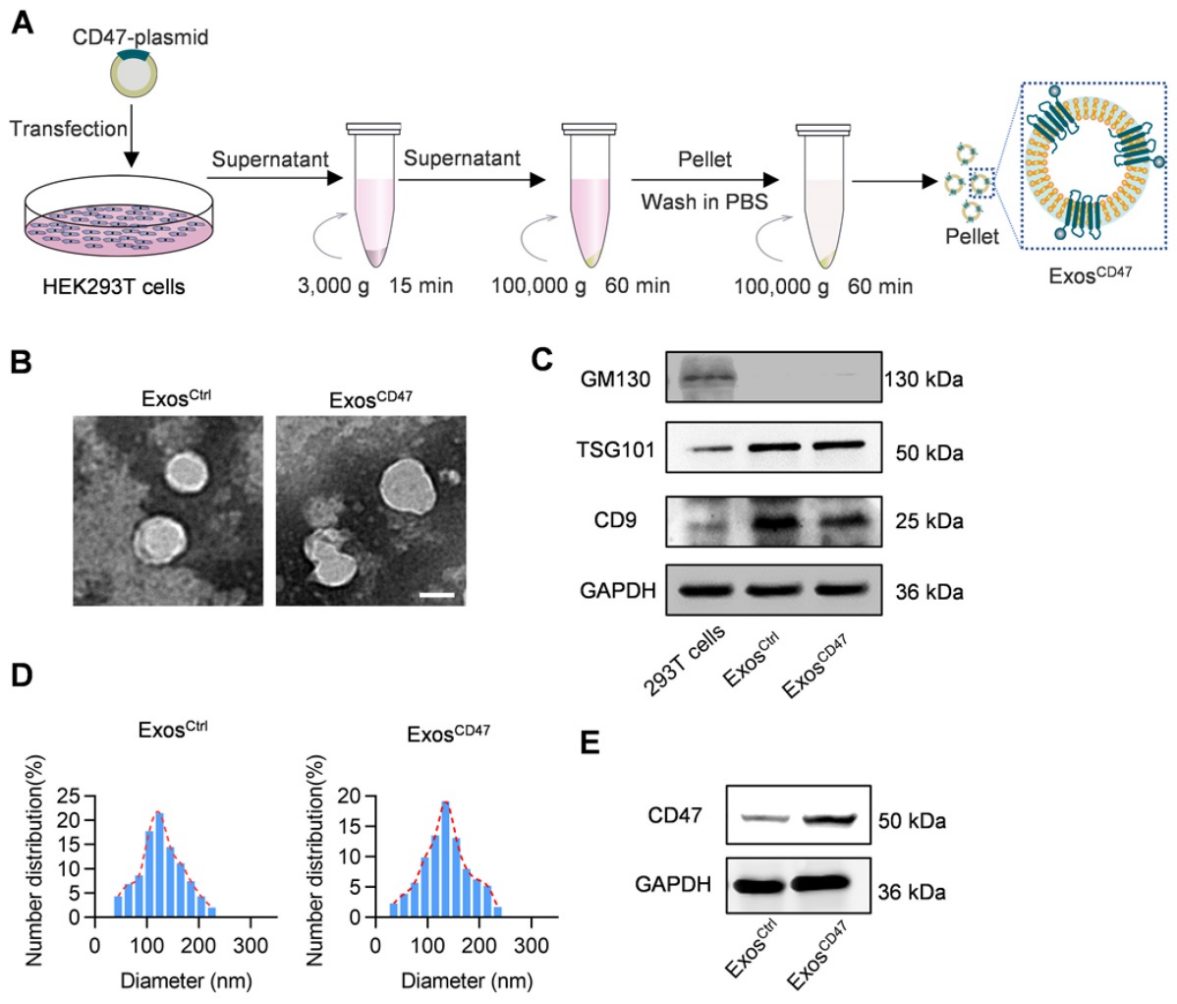
** Preparation and characterization of Exos^CD47^.** (A) Schematic illustration of exosomes preparation. (B) Representative images of transmission electron micrographs of Exos^Ctrl^ and Exos^CD47^. Scale bar = 100 nm, n = 3 samples per group. (C) Western blot analysis of exosome markers TSG101, CD9 and exclusive exosomal marker GM130 on 293T cells, Exos^Ctrl^ and Exos^CD47^. GAPDH served as internal control. (D) Size distribution of exosomes Exos^Ctrl^ and Exos^CD47^. n = 3 samples per group. (E) Western blot analysis of CD47 on exosomes (Exos^Ctrl^ and Exos^CD47^). GAPDH served as internal control.

To functionalize the exosomes with CD47 on the surface, the donor cells (HEK293T) were transfected with CD47 overexpressing plasmid (Figure 1A). Transmission electron microscope analysis revealed that the isolated vesicles showed similar morphology as the typical exosomes (Figure 1B). Western blot analysis showed that there were abundant TSG101 and CD9 in both Exos^Ctrl^ and Exos^CD47^. In contrast, the Golgi complex marker GM130 was not found in the isolated exosomes (Figure 1C). Moreover, the expression of CD47 from Exos^CD47^ increased significantly in comparison with the Exos^Ctrl^ (Figure 1E). To further confirm whether CD47 was expressed on the exosomal surface, the exosomes were captured by anti-CD47, followed by western blot analysis of CD9 exosomal marker. As expected, anti-CD47 could capture the exosomes, suggesting that CD47 was mainly expressed on the exosome membrane (Figure S1). As shown in Figure 1D, the diameter of Exos^CD47^ ranged from 30 to 150 nm, which was indistinguishable from typical exosomes. These data confirmed the exosome feature of Exos^CD47^.

### Enhanced tumor targeting of Exos^CD47^ via mononuclear phagocytic system escape

**Figure 2:**
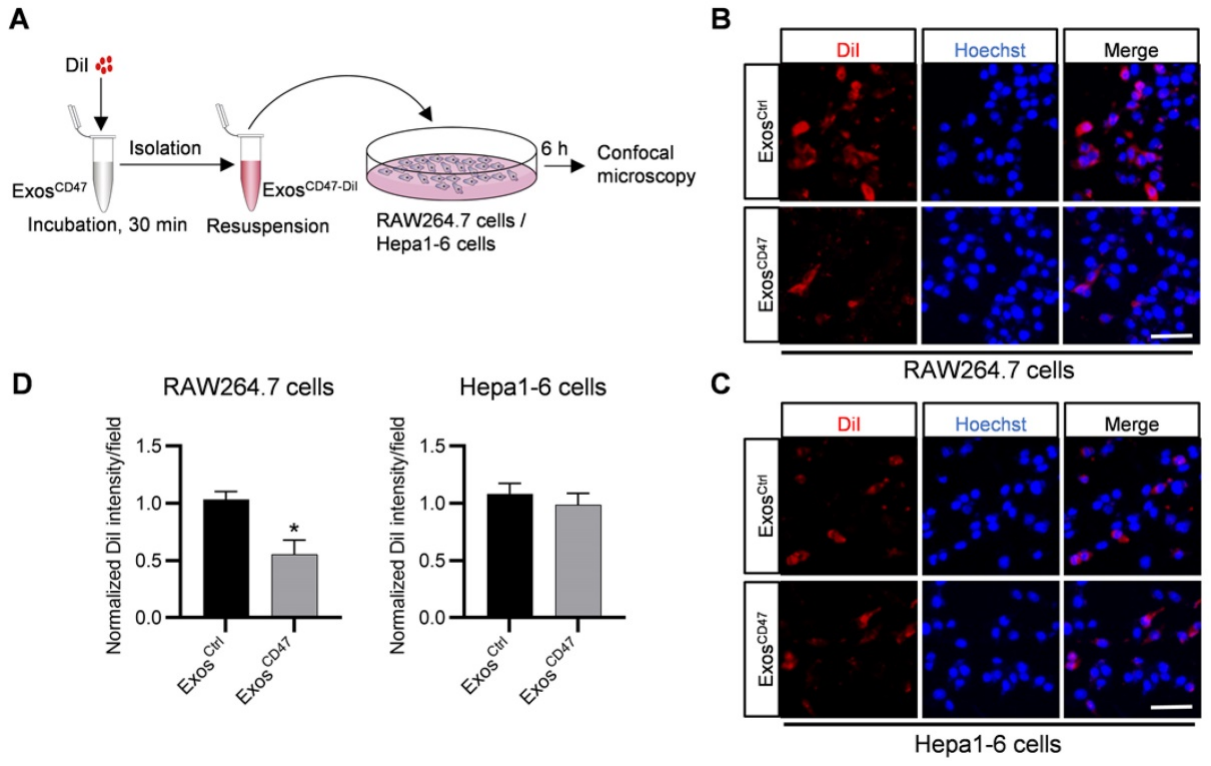
** Exos^CD47^ can escape the phagocytosis from macrophages.** (A) Schematic illustration of the experiment. (B-C) Representative confocal fluorescence images of the DiI-labeled exosomes (red) in RAW264.7 cells and Hepa1-6 cells. The nuclei were counter-stained with Hoechst (blue). Scale bar = 20 μm, n = 3 samples per group. (D) Quantification of fluorescence intensity of the DiI-labeled exosomes in RAW264.7 cells and Hepa1-6 cells. n = 3, *, *P* < 0.05.

In order to explore whether Exos^CD47^ can escape the phagocytosis of the MPS and increase tumor targeting specificity, DiI-labeled Exos^Ctrl^ and Exos^CD47^ were incubated with RAW264.7 cells and Hepa1-6 cells respectively (Figure 2A). As expected, *in vitro* fluorescence imaging showed that the amount of Exos^CD47^ phagocytosed by RAW264.7 cells was much less than the Exos^Ctrl^ (Figure 2B-D). However, there were no significant differences of the phagocytosis efficiency by tumor cells between Exos^Ctrl^ and Exos^CD47^ (Figure 2C-D). In addition, qPCR tracing of the cel-miR-54-5p encapsulated in the exosomes was also applied to verify these results (Figure S2A). Exos^Ctrl^ and Exos^CD47^ were loaded with same amount of cel-miR-54-5p mimics by electroporation, followed by incubation with RAW264.7 and Hepa1-6 cells (Figure S2A-B). As expected, the expression of cel-miR-54-5p in RAW264.7 in Exos^CD47^ group was notably less than that in Exos^Ctrl^ group (Figure S2C). Moreover, there was no difference of cel-miR-54-5p in Hepa1-6 cells (Figure S2D).

**Figure 3:**
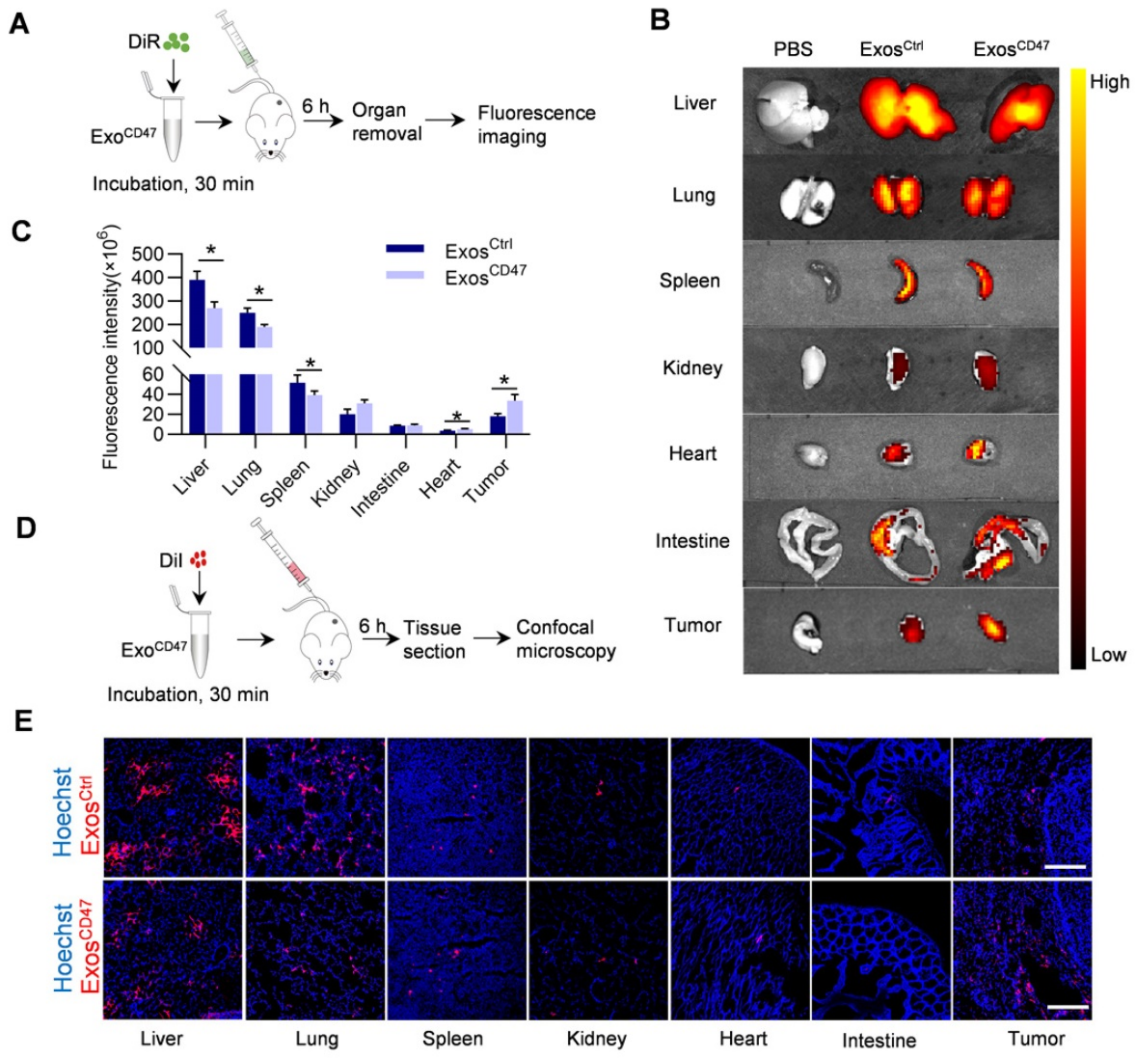
** Exos^CD47^ escape from MPS *in vivo*.** (A) Schematic illustration of the experimental procedure. (B) Representative ex vivo fluorescent images of the DiR-labeled exosomes in tumors and main organs. n = 5 mice. (C) Quantification of fluorescence intensity of different organs and tumors. Data are presented as the mean ± SEM, n = 5, *, *P* < 0.05. (D) Schematic illustration of the experimental procedure. (E) Representative fluorescence images of the DiI-labeled exosomes (red) in tumors and main organs. The nuclei were counter-stained with Hoechst (blue). Scale bar = 100 μm, n = 5 mice.

In order to detect the targeted delivery of Exos^CD47^ in target organs, DiI-labeled or DiR-labeled Exos were injected into the mice via the tail vein. Compared with Exos^Ctrl^, the distribution of Exos^CD47^ in the livers, lungs and spleens was significantly reduced, and the distribution in the intestines and kidneys was slightly increased. Most importantly, the distribution in tumors was increased, as revealed by ex vivo imaging (Figure 3A-C). Consistent with the ex vivo fluorescent imaging data, confocal imaging analysis of organ tissue sections showed similar results (Figure 3D-E). In addition, the results of Quantitative real-time PCR showed that Exos^CD47^ had longer circulation time than Exos^Ctrl^ (Figure S2A, Figure S2E).

### Encapsulation of Er and RB into exosomes via sonication

**Figure 4:**
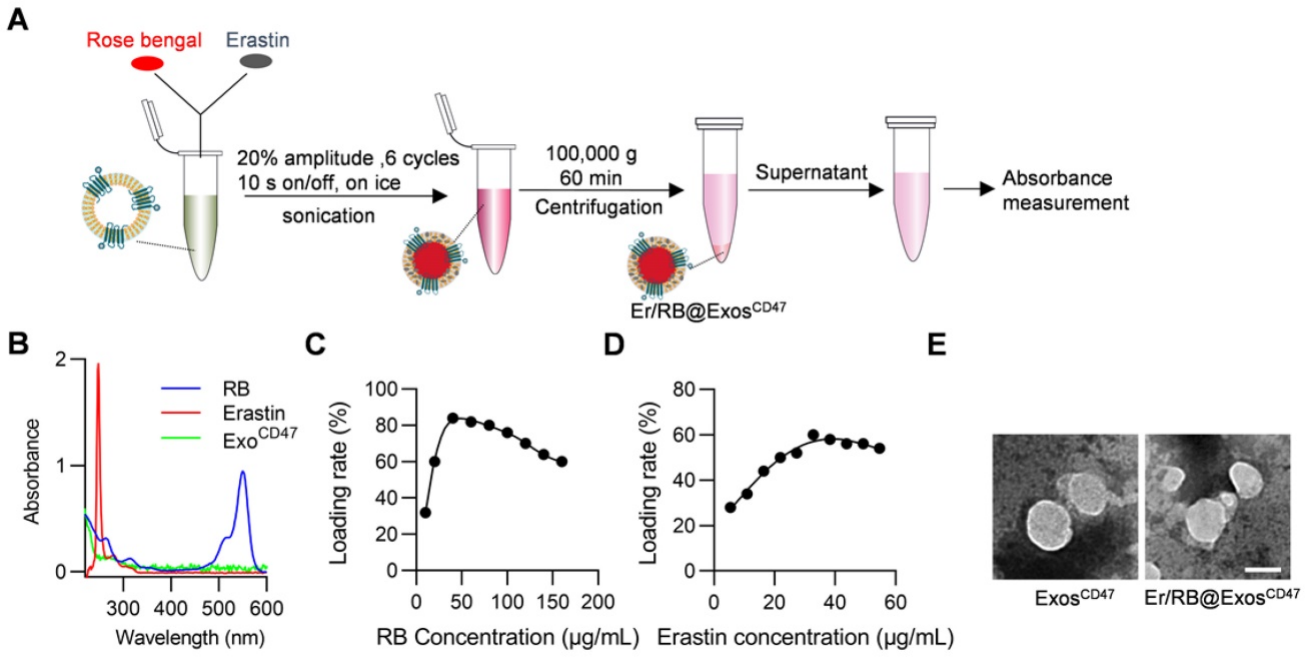
** Efficient loading of Erastin and RB into exosomes.** (A) Schematic illustration of the procedure how Erastin and RB are loaded into exosomes. (B) UV spectroscopy of RB and Erastin in PBS, the maximum UV absorption peak of RB is at 550 nm and Erastin at 249 nm. (C-D) Different amount of RB/Erastin was respectively added to Exos^CD47^ (600 μg/mL), the curves show the relationship between the loading rate of RB or Erastin in Exos^CD47^ and its concentration. (E) Representative images of transmission electron micrographs of Exo^CD47^, Er/RB@Exo^CD47^. Scale bar = 100 nm, n = 3 samples per group.

We next encapsulate Er/RB into the exosomes via sonication (Figure 4A). Er and RB concentrations were determined by the absorption measurement. The maximum absorption peaks of Er and RB in PBS are 249 nm and 550 nm, respectively (Figure 4B). Within a certain concentration range, the results (Figure S3) showed that the intensity and concentration of the maximum absorption peak were linearly related, so we used the intensity of the maximum absorption peak to calculate the concentration of the drugs. When the exosomes at the concentration of 600 μg/mL, the maximum encapsulation rates of RB and Er reached 84% and 60%, respectively. In other words, 32.8 μg Er was loaded per mg exosomes, while the 56 μg RB was loaded per mg exosomes. Importantly, the morphology of the exosomes had no significant change after sonication, as observed by transmission electron microscope (Figure 4E).

### Er/RB@Exos^CD47^ together with laser irradiation induce ferroptosis in Hepa1-6 cells

**Figure 5:**
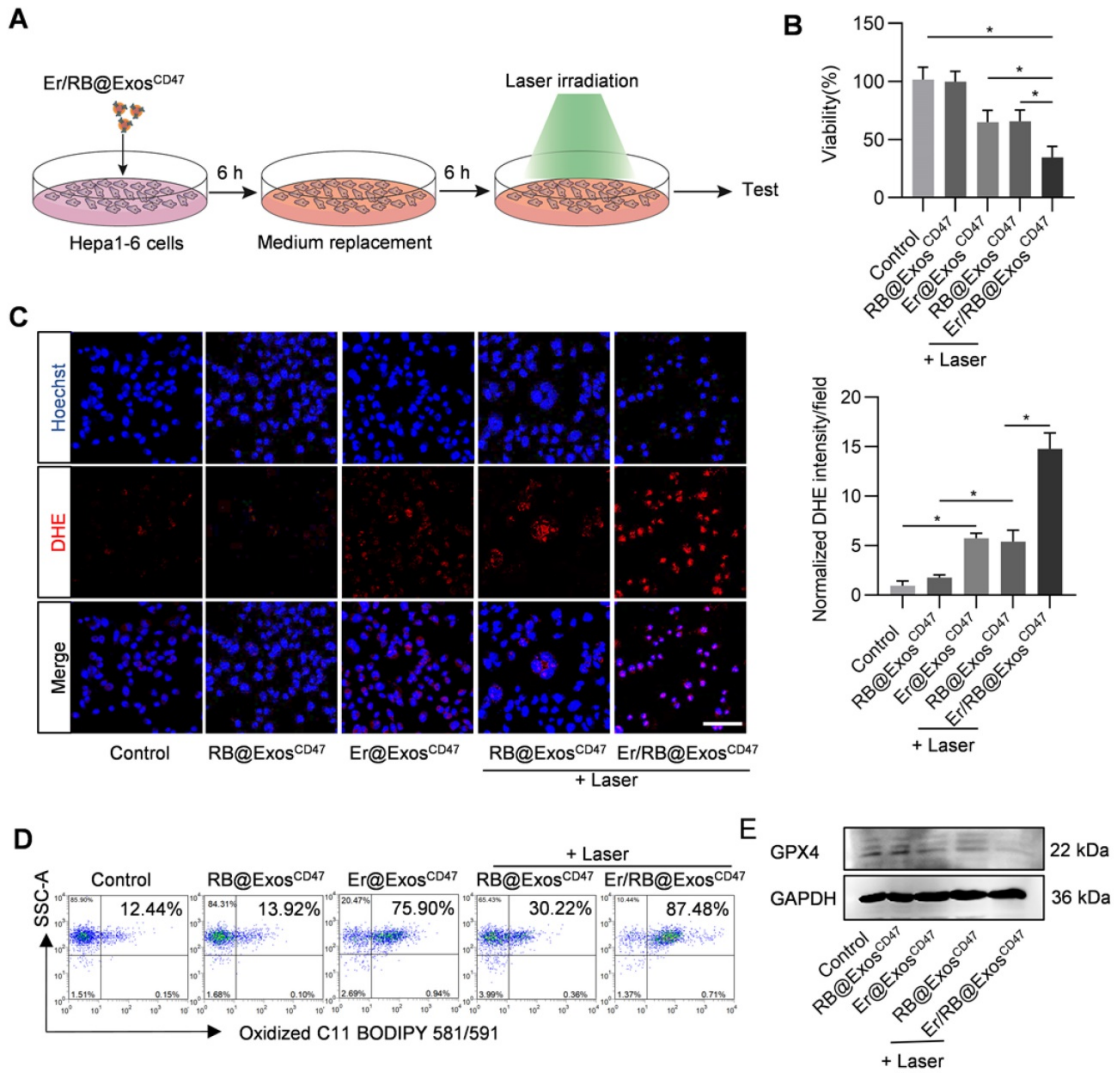
** Ferroptosis induction by Er/RB@Exos^CD47^ combined with PDT.** (A) Schematic illustration of the experimental procedure. (B) Cell viability detection by MTT assay. Data are presented as the mean ± SEM, n = 3, *, *P* < 0.05. (C) Total ROS generation in Hepa1-6 cells after different treatments was detected by fluorescence microscopy, Scale bar = 50 μm. Data are presented as the mean ± SEM, n = 3, *, *P* < 0.05. (D) Lipid ROS of Hepa1-6 cells detected by flow cytometry. Representative data of three different experiments. (E) Western blot analysis of GPX4 in Hepa1-6 cells after treated with different formulations. GAPDH served as internal control.

In order to verify the therapeutic effect of Er/RB@Exos^CD47^, Hepa1-6 cells were treated with Control, RB@Exos^CD47^, Er@Exos^CD47^, RB@Exos^CD47^(L) and Er/RB@Exos^CD47^ (L) (Figure 5A). Er/RB@Exos^CD47^ (L) significantly induce cell death (Figure 5B). To further confirm whether Er/RB@Exos^CD47^ (L) induce cell death in the form of ferroptosis, lipid ROS and GPX4 expression were analyzed. As expected, Er@Exos^CD47^ and RB@Exos^CD47^(L) treatment moderately increased the ROS (Figure 5C), while the level of ROS in Hepa1-6 cells treated with Er/RB@Exos^CD47^(L) was significantly higher than that in all other groups. Notably, photosensitizer RB alone had no effect on ROS level of Hepa1-6 cells. Similar results were observed by flow cytometry analysis of C11 BODIPY 581/591 (Figure 5D, Figure S4). Consistent with the increased ROS, GPX4 expression was obviously downregulated by Er@Exos^CD47^and Er/RB@Exos^CD47^(L) (Figure 5E, Figure S4). These data indicated that Er/RB@Exos^CD47^(L) could significantly induce tumor cells ferroptosis and RB@Exos^CD47^(L) could induce ferroptosis by accumulation of lipid ROS not downregulating the GPX4 expression.

### Therapeutic effects of Er/RB@Exos^CD47^ together with PDT in HCC xenograft model

**Figure 6:**
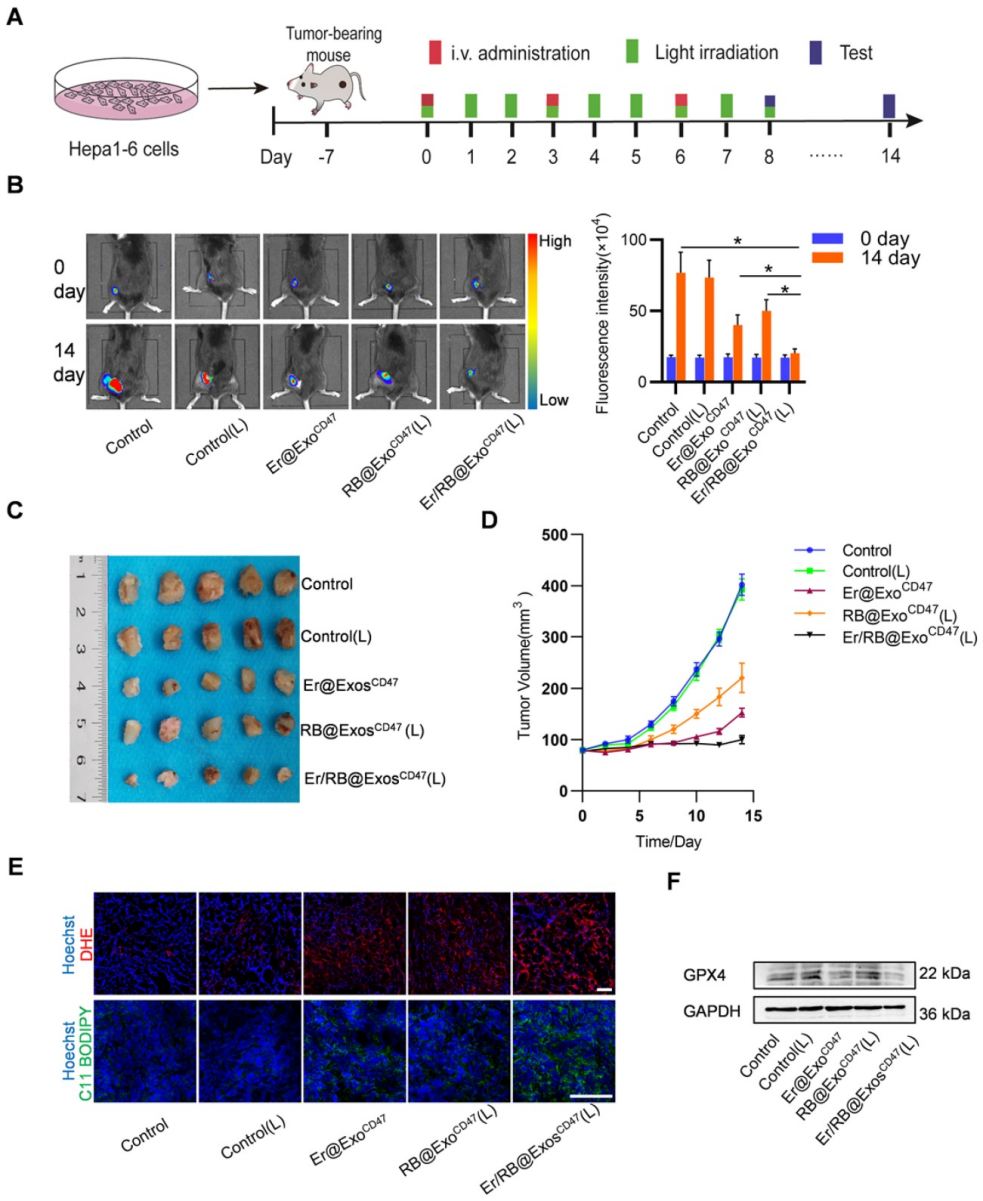
** Therapeutic efficacy of Er/RB@Exos^CD47^ combined with PDT *in vivo*.** (A) Schematic illustration of the experimental procedure. (B) *In vivo* representative fluorescence imaging of subcutaneous Hepa1-6-luc tumor-bearing C57BL/6 mice with different treatments. Data are presented as the mean ± SEM, n = 5, *, *P* < 0.05. (C) Tumor images of mice with different treatment for 14 days. (D) Tumor growth curves of tumor volume after different treatment for 14 days. (E) Representative images of DHE and C11 BODIPY 581/591 staining of tumor sections. Scale bar = 25 μm. n = 5 mice. (F) Western blot analysis of GPX4 in tumors after treated with different formulations. GAPDH served as internal control.

In the following experiments, we explored the therapeutic effect of Er/RB@Exos^CD47^ combined with laser irradiation in the HCC xenograft model, in which Hepa1-6-luc cells were used. When the tumors reached 80 mm^3^, the tumor-bearing mice were divided into 5 groups: Control, Control (L), Er@Exos^CD47^, RB@Exos^CD47^ (L) and Er/RB@Exos^CD47^ (L). Exosomes were injected via tail vein every three days, and green laser light irradiation was administered for 15 min in the laser groups twice a day (Figure 6A). After 14 days treatment, the tumor fluorescence signal of the Er/RB@Exos^CD47^ (L) was the weakest, while the Er@Exo^CD47^ and RB@Exos^CD47^(L) was moderately lowered in comparison with Control and Control (L) group (Figure 6B). Accordingly, the tumor volume of in the Er/RB@Exo^CD47^ (L) group was the smallest (Figure 6C-D).

Consistent with the tumor growth inhibition in Er/RB@Exo^CD47^ (L) group, the total ROS and lipid ROS in tumors of Er/RB@Exo^CD47^ (L) group was the highest (Figure 6E), suggesting the most potent ferroptosis induction in the group. In addition, GPX4 expression of tumors was evaluated by western blotting. GPX4 expression was obviously downregulated in Er/RB@Exos^CD47^(L) group, moderately Er/RB@Exos^CD47^ and slightly RB@Exos^CD47^(L) compared to Control group (Figure 6F). The results were consistent with that *in vitro*. The cytotoxicity of Er/RB@Exos^CD47^ was also evaluated. As shown in Figure S5A. there was much obvious side-effects in liver Er/RB@Exos^Ctrl^ treated group, as seen from HE staining (Figure S5A) and biochemistry analysis (Figure S5B-D).

## Discussion

In this study, we successfully constructed CD47-functionalized exosomes that could evade MPS and enhance tumor targeting. In addition, we used sonication to efficiently load Er and RB into exosomes, and *in vivo* and *in vitro* experiments have verified that it could promote the ferroptosis process of tumors and play anti-tumor effect.

In our research, we used the strategy of inducing ferroptosis to kill tumors. Existing evidence has proved that the method of inducing ferroptosis is to effectively kill cancer cells through the accumulation of ROS in the cells and is iron dependent 10. It has been reported that gene inactivation or drug inhibition of GPX4 can promote cells ferroptosis 40, 41. This strategy enhanced ferroptosis by reducing the degradation of ROS and increasing its accumulation in cells. However, GPX4 gene ablation resulted in increased accumulation of oxidized phospholipid products in the kidney, leading to AKI and early death in mice 42. Furthermore, there have been many recent studies 43-45 that used iron-based nanomaterials as ferroptosis-inducing agents to increase iron supply and enhance ferroptosis to achieve the effect of treating tumors. Unfortunately, these methods could not solve the problem of the harmful toxicity of the vector and the low ROS conversion efficiency.

Herein, we adopted the strategy of ferroptosis inducer-Er combined with PDT to amplify the effect of ferroptosis in tumor treatment. In PDT, molecular oxygen can be converted into reactive oxygen species (ROS) by photosensitizers (PSs) in their respective excited states 46. We used the large amount of ROS produced in PDT to supplement the accumulation of lipid ROS required for ferroptosis, thereby enhancing the therapeutic effect of ferroptosis. In our research, *in vitro* and *in vivo* experiments have fully verified our research assumption. ROS has a short lifespan in biological systems, and the diffusion radius of its generation site (< 30 nm) is limited 47, so PDT selectively targets the position of PS during light irradiation. Therefore, our strategy of combined PDT and chemotherapy could induce efficient ferroptosis in tumors with low dose of drugs, which could not only reduce side effects, but also avoid the biological safety issues of knocking out and inactivating GPX4 in the previous research methods, as well as the low ROS conversion rate and carrier toxicity issues in the iron overloading research method.

Previous studies have shown that exosome was an effective biological carrier for drugs for therapeutic purposes 48. However, a major obstacle to exosome-based treatment was its phagocytosis by MPS, which limited its clinical application 49. Therefore, in our study, we designed Exos^CD47^ as a delivery vehicle for these two drugs. It has been widely reported that CD47 was mainly expressed on the surface of circulating hematopoietic stem cells 36, normal erythrocytes 50 and most types of tumor cells 51. CD47 was a transmembrane molecule which bound to SIRPα on the surface of macrophages to produce “don't eat me” signal to prevent phagocytosis 52. Several drug delivery nanocarriers have been used in cancer treatment, but they have certain limitations. For example, nanoparticles have a limited load capacity, which is less than 5%. Another major problem is that the payload will be released suddenly before reaching the predetermined position 22. Another drug delivery carrier, gold nanoparticles have problems such as biodistribution and cationic ligand toxicity 23. The Exos^CD47^ that we successfully constructed have been verified *in vivo* distribution experiments to evade the phagocytosis of MPS, and increase the distribution in the tumor. And through our experimental verification, the encapsulation rate of each drug in this bio-delivery carrier had reached 60%. In the experiment, we used CD47-overexpressed exosomes to load both Er and RB at the same time instead of simply loading one drug. On the one hand, this experimental design can better exert the superimposed effect of the combination of the two methods. On the other hand, the operability is more convenient and safer.

Although our treatment method had a good therapeutic effect on tumors *in vitro* and *in vivo*, we still need to conduct further experimental research in the future. For example, according to the current technology, it is not possible to prepare exosomes in large quantities. In view of this, exploring new technologies to generate exosomes in sufficient quantities will be a major research project in our future.

In conclusion, we have engineered an exosome armed with three moieties, surface functionalization with CD47, membrane loading with ferroptosis inducer Er, and core with photosensitizer RB. By using the HCC xenograft model, we here showed that the designed exosomes display potent anti-tumor therapeutic effects, and amazing low toxicity. Thus, the engineered exosomes would provide a promising strategy to treat malignant tumors.

## Supplementary Material

Supplementary figures and table.
