# Supplementary material for: Designer exosomes for targeted and efficient ferroptosis induction in cancer via chemo-photodynamic therapy
Source: Theranostics. 2021 Jul 13;11(17):8185–96. doi: 10.7150/thno.59121 (PMC8344009; doi:10.7150/thno.59121)
Supplement: Supplementary file 1 — Supplementary figures and table. [file thnov11p8185s1.pdf]

Supporting Information

Designer exosomes for targeted and efficient ferroptosis induction in cancer via chemo-photodynamic therapy

Jianbing Du[1,2#], Zhuo Wan[3#], Cong Wang[4#], Fan Lu[5], Mengying Wei[5], Desheng Wang[1]*, Qiang Hao[2]*

1. Department of Hepatobiliary Surgery, Xijing Hospital, Fourth Military Medical University, Xi'an, 710032, China.

2. State Key Laboratory of Cancer Biology, Biotechnology Center, School of Pharmacy, Fourth Military Medical University, Xi'an, 710032, China.

3. Department of Hematology, Tangdu Hospital, Fourth Military Medical University, Xi'an, 710038, People's Republic of China.

4. Department of Clinical Laboratory, The Second People's Hospital of Hefei, Hefei, China.

5. State Key Laboratory of Cancer Biology, Department of Biochemistry and Molecular Biology, Fourth Military Medical University, Xi'an, 710032, People's Republic of China.

# These authors contributed equally to this article.

* Correspondence authors:

E-mail addresses:

wangdesh@163.com(Desheng Wang)

haosuq@fmmu.edu.cn(Qiang Hao)

Table S1. Primers and miRNA sequences used in the study

PCR primer	Forward (5'-3')	Reverse (5'-3')
U6	CTCGCTTCGGCAGCACA	AACGCTTCACGAATTTGCGT
cel-miR-54-5p	AGGATATGAGACGACG AGAACA	Provided in the kit
Mimics	Sequence (5'-3')	
cel-miR-54-5p mimics	AGGAUAUGAGACGACGAGAACA	

Supplementary figures and figure legends

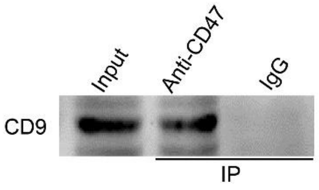

Figure S1. Surface expression of CD47 on the exosomes. Exosomes from CD47 expressing cells were isolated and further pulled down by anti-CD47, IgG. Exosomes were successfully pulled down, suggesting that CD47 expressed at the membrane of exosomes. Representative image of three different experiments.

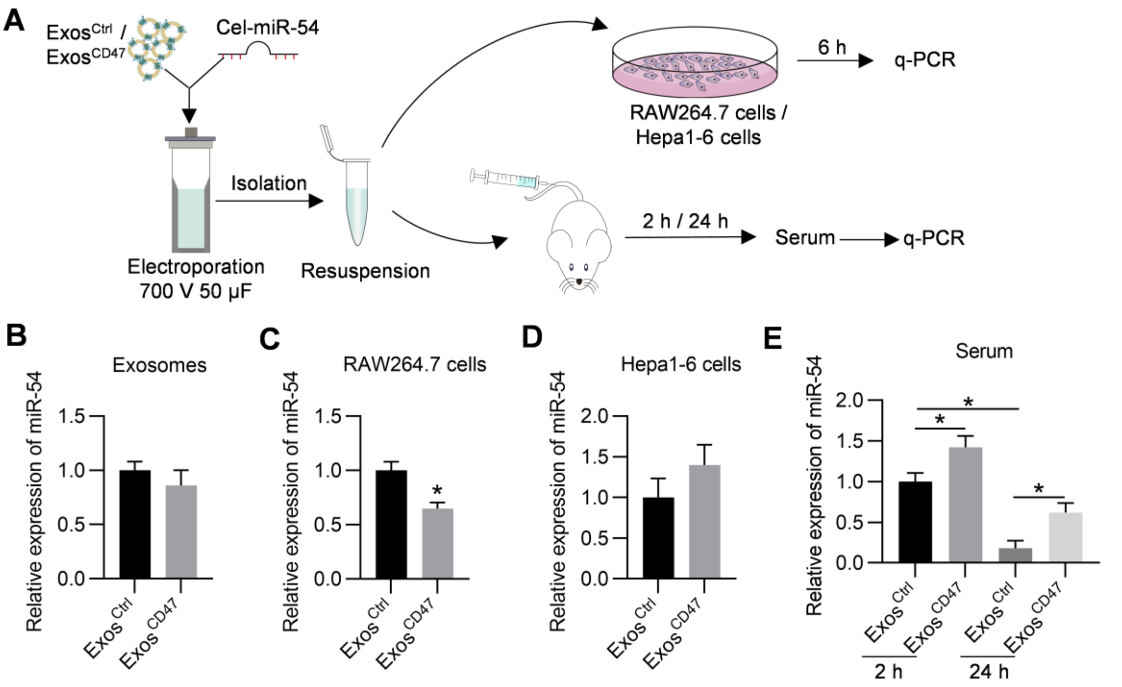

Figure S2. Effects of CD47 engineering on endocytosis and circulation of exosomes. (A) Schematic illustration of the experiment. Exosomes were loaded with cel-miR-54 via electroporation. (B) qPCR analysis of miR-54 in $Exos^{Ctrl}$ and $Exos^{CD47}$. U6 served as internal control. Data are presented as the mean ± SEM, n = 3 samples per group. (C-D) qPCR analysis of miR-54 in RAW264.7 cells and Hepa1-6 cells. U6 served as internal control, data are presented as the mean ± SEM, n = 3 samples per group. *, $P < 0.05$. (E) qPCR analysis of miR-54 in serum. U6 served as internal control, data are presented as the mean ± SEM, n = 5 mice per group. *, $P < 0.05$.

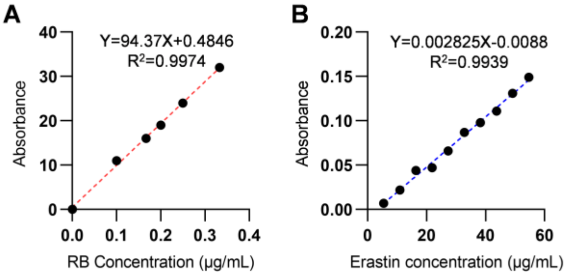

Figure S3. Absorbance curve of Erastin and RB in PBS. The graphs show the linear relationship between absorbance at the max UV absorption and concentration of RB (A) and Erastin (B).

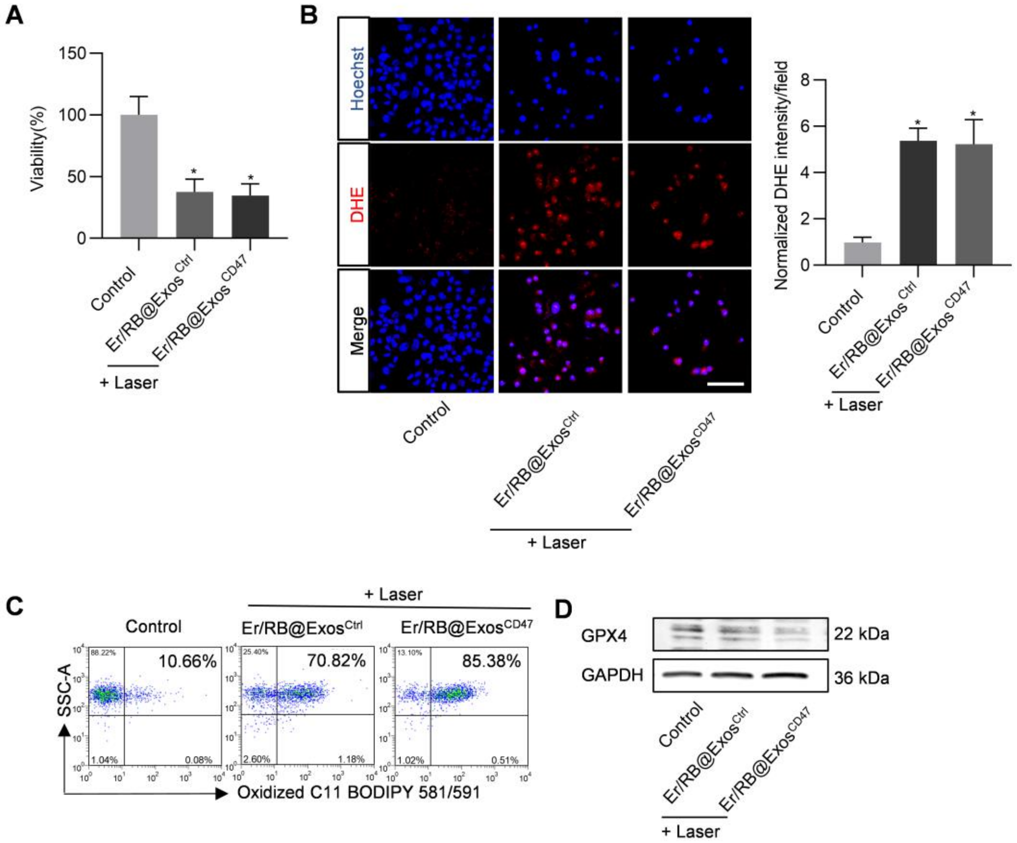

Figure S4. Effective ferroptosis induction by Er/RB@Exo^{CD47} with laser irradiation. (A) Cell viability detection by MTT assay. Data are presented as the mean ± SEM, n = 3 samples per group, *, $P < 0.05$. (B) ROS generation in Hepa1-6 cells after different treatments was detected by fluorescence microscopy. Scale bar = 50 μm. Data are presented as the mean ± SEM, n = 3 samples per group, *, $P < 0.05$. (C) Lipid ROS of Hepa1-6 cells detected by flow cytometry. Representative data of three different experiments. (D) Western blot analysis of GPX4 in Hepa1-6 cells after treated with

different formulations. GAPDH served as internal control.

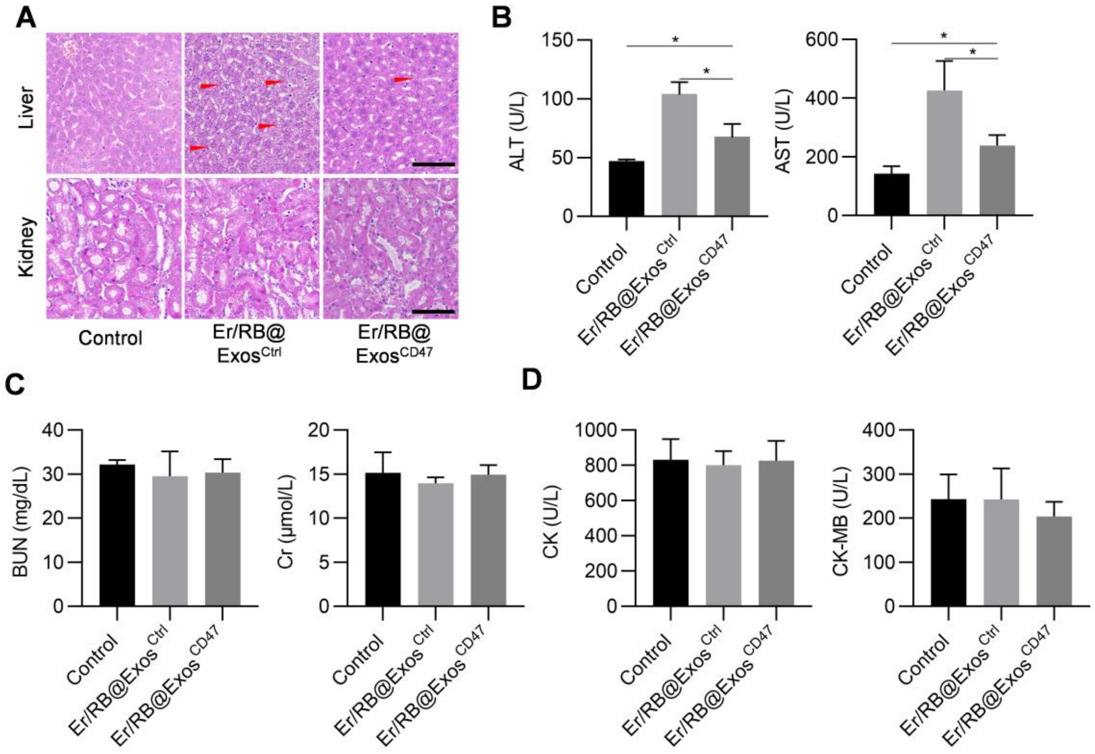

Figure S5. Off-target effects of control and Er/RB@Exos^{CD47}. (A) H&E images of liver and kidney tissue of mice treated with different formulations. The red arrows indicate the site of lobular inflammation. Scale bar = 100 μm. n = 5 mice. (B) Liver toxicity test, consisting of alanine transaminase (ALT) and aspartate transaminase (AST) test in the listed groups. Data were expressed as mean ± SEM, n = 5, *, $P < 0.05$. (C) Kidney toxicity test, consisting of blood urea nitrogen (BUN) and creatinine (Cr) test. Data were expressed as mean ± SEM, n = 5. (D) Heart toxicity test, consisting of creatine kinase (CK) and creatine kinase-MB (CK-MB) test. Data were expressed as mean ± SEM, n = 5.
